# The CLE53–SUNN genetic pathway negatively regulates arbuscular mycorrhiza root colonization in *Medicago truncatula*

**DOI:** 10.1093/jxb/eraa193

**Published:** 2020-04-20

**Authors:** Magda Karlo, Clarissa Boschiero, Katrine Gram Landerslev, Gonzalo Sancho Blanco, Jiangqi Wen, Kirankumar S Mysore, Xinbin Dai, Patrick X Zhao, Thomas C de Bang

**Affiliations:** 1 Department of Plant and Environmental Sciences, Faculty of Science, University of Copenhagen, Frederiksberg C, Denmark; 2 Noble Research Institute LLC, Ardmore, OK, USA; 3 Lancaster University, UK

**Keywords:** Autoregulation, CLE peptides, *Medicago truncatula*, mycorrhiza, RDN1, SUNN

## Abstract

Plants and arbuscular mycorrhizal fungi (AMF) engage in mutually beneficial symbioses based on a reciprocal exchange of nutrients. The beneficial character of the symbiosis is maintained through a mechanism called autoregulation of mycorrhization (AOM). AOM includes root-to-shoot-to-root signaling; however, the molecular details of AOM are poorly understood. AOM shares many features of autoregulation of nodulation (AON) where several genes are known, including the receptor-like kinase SUPER NUMERIC NODULES (SUNN), root-to-shoot mobile CLAVATA3/ENDOSPERM SURROUNDING REGION (ESR)-RELATED (CLE) peptides, and the hydroxyproline *O*-arabinosyltransferase ROOT DETERMINED NODULATION1 (RDN1) required for post-translational peptide modification. In this work, CLE53 was identified to negatively regulate AMF symbiosis in a SUNN- and RDN1-dependent manner. CLE53 expression was repressed at low phosphorus, while it was induced by AMF colonization and high phosphorus. CLE53 overexpression reduced AMF colonization in a SUNN- and RDN1 dependent manner, while *cle53*, *rdn1*, and *sunn* mutants were more colonized than the wild type. RNA-sequencing identified 700 genes with SUNN-dependent regulation in AMF-colonized plants, providing a resource for future identification of additional AOM genes. Disruption of AOM genes in crops potentially constitutes a novel route for improving AMF-derived phosphorus uptake in agricultural systems with high phosphorus levels.

## Teaser

CLE53 negatively regulates root colonization with arbuscular mycorrhizal fungi in a SUNN- and RDN1-dependent manner.

## Introduction

Arbuscular mycorrhizal fungi (AMF) and plants engage in a mutually beneficial symbiosis based on a reciprocal exchange of nutrients. Plants provide carbohydrates in the form of hexoses (Pfeffer *et al.*, 1999) and lipids (Jiang *et al.*, 2017; Luginbuehl *et al.*, 2017) to the AMF, which in return provide mineral nutrients obtained through extended hyphal networks in the soil. AMF particularly improve plant uptake of inorganic phosphate (Pi) in both natural and agricultural ecosystems (Smith *et al.*, 2011). The majority of all crops engage in AMF symbiosis, and hence understanding the mechanisms regulating plant–AMF interactions constitutes a means to improve phosphorus use efficiency in agriculture. AMF function as strong carbon sinks and up to 20% of plant photosynthates are spent on maintaining the symbiosis (Jakobsen and Rosendahl, 1990; Bago *et al.*, 2000). To regulate carbon drainage and thus maintain a beneficial AMF symbiosis, plants adjust AMF colonization through a systemic mechanism known as autoregulation of mycorrhization (AOM). AOM depends on root-to-shoot-to-root signaling, where an initial signal produced in the root upon AMF colonization functions to limit subsequent colonization events (Vierheilig *et al.*, 2000; Catford *et al.*, 2003). However, the underlying molecular mechanisms of AOM are poorly understood.

A mode of systemic regulation, very similar to AOM, occurs in response to symbiotic interaction between leguminous plants and nitrogen-fixing soil bacteria collectively known as rhizobia. Rhizobia are hosted in special root organs called nodules where they fix and convert atmospheric di-nitrogen into ammonia, which is provided to the host plant in exchange for carbohydrates. Similarly to AOM, legumes strictly control the formation of nodules and thereby the carbon costs associated with rhizobial nitrogen fixation through a systemic root-to-shoot-to-root regulatory feedback mechanisms known as autoregulation of nodulation (AON) (Kosslak and Bohlool, 1984; Reid *et al.*, 2011*a*). AON is activated immediately after rhizobial infection via production of nodulation-specific plant peptides from the CLAVATA3/Endosperm Surrounding Region (ESR)-related (CLE) family (Mortier *et al.*, 2010; Okamoto *et al.*, 2013). The root-derived CLE peptides travel via the xylem to the shoot and activate a signaling pathway through interaction with a leucine-rich repeat receptor-like kinase (LRR-RLK) highly similar to CLAVATA1 from *Arabidopsis thaliana* (Okamoto *et al.*, 2013). Mutant alleles in this receptor have been identified in several legume plant species including *har1* in *Lotus japonicus* (Krusell *et al.*, 2002; Nishimura *et al.*, 2002), *sym29* in pea (Krusell *et al.*, 2002), *nark* in soybean (Searle *et al.*, 2003), and *sunn* in *Medicago truncatula* (Penmetsa *et al.*, 2003; Schnabel *et al.*, 2005). The receptor mutants are all defective in AON and exhibit varying degrees of uncontrolled nodule formation termed supernodulation or hypernodulation. Known molecular players downstream of HAR1/SUNN include the Kelch-repeat F-box protein TOO MUCH LOVE (TML) (Takahara *et al.*, 2013; Gautrat *et al.*, 2019) and the miRNA, miR2111 that, together with cytokinin, functions as part of the shoot-to-root signaling pathway (Sasaki *et al.* 2014; Tsikou *et al.*, 2018). In addition, a second AON pathway independent of SUNN was recently proposed, adding to the complexity of autoregulation (Kassaw *et al.*, 2015). Some of the above-mentioned AON mutants have also been shown to be defective in AOM, though the associated increase in AMF colonization is smaller than the increase in nodule numbers observed in the AON mutants (Morandi *et al.*, 2000; Shrihari *et al.*, 2000; Meixner *et al.*, 2005; Sakamoto and Nohara 2009). Furthermore, the LRR receptor CLAVATA2, which is also known to act in AON, was recently shown to be part of AOM in tomato (Wang *et al.*, 2018). This suggests that the AON and AOM pathways are highly similar and possibly initiated by a similar signal ascending from root-to-shoot, namely a CLE peptide, as suggested previously (Staehelin *et al.*, 2011; de Bang *et al.*, 2017*a*; Wang *et al.*, 2018; Müller and Harrison, 2019).

CLE peptides control many important developmental processes in plants such as meristem maintenance and root development, and are involved in response to environmental cues such as nutrient deficiencies and drought (Yamaguchi *et al.*, 2016; de Bang *et al.*, 2017*b*; Takahashi *et al.*, 2018). CLEs are post-translationally modified small signaling peptides of 12–13 amino acids that act as extracellular ligands for cell surface LRR-RLK receptors (Matsubayashi, 2014). Bioactive CLEs are derived from larger non-functional pre-proproteins of ~100 amino acids in size and are encoded in the genomes of all seed plants, as well as in lycophytes and mosses, but not in green algae (Goad *et al.*, 2017). CLE peptide sequences are highly conserved across species, and two proline residues constitute the main targets of post-translational modification. Prolines of AON CLE peptides are converted to hydroxyprolines (Hyps) and further modified with a chain of three l-arabinose moieties (Ohyama *et al.*, 2009; Okamoto *et al.*, 2013). This modification is critical for receptor perception and thus biological activity (Shinohara and Matsubayashi, 2013; Corcilius *et al.*, 2017; Imin *et al.*, 2018; Hastwell *et al.*, 2019). Hydroxyproline *O*-arabinosyltransferases (HPATs) catalyze the transfer of l-arabinose to the hydroxyl group of Hyp residues (Xu *et al.*, 2015). *Medicago truncatula* and pea mutants of the functional HPAT homologs *ROOT DETERMINED NODULATION1* (*RDN1*) and *NOD3* displayed supernodulation phenotypes (Schnabel *et al.*, 2011). MtRDN1 is required for the function in AON of nodulation-specific MtCLE12, but not MtCLE13 (Kassaw *et al.*, 2017), which was experimentally supported by arabinosylated synthetic MtCLE12 and MtCLE13 peptides (Imin *et al.*, 2018).

In *L. japonicus*, expression of the two CLE peptides LjCLE19 and LjCLE20 was induced in response to phosphorus (P) (Funayama-Noguchi *et al.*, 2011) and, together with four other CLE peptides, they were also induced in mycorrhizal plants (Handa *et al.*, 2015). Most recently, and in parallel to this work, Müller *et al.* (2019) showed that ectopic overexpression of both the AMF-induced *MtCLE53* and the P-induced *MtCLE33* was able to repress AMF colonization in a SUNN-dependent manner. The repression of AMF colonization was linked to a reduced production of strigolactones, a class of phytohormones known to stimulate initiation of the AMF symbiosis (Akiyama *et al.*, 2005) and to be required for secondary hyphal infections (Kobae *et al.*, 2018).

We here show that the expression of *MtCLE53* is induced in roots in a local response to AMF and P, and that MtCLE53 acts as a negative regulator of AMF colonization in a SUNN- and RDN1-dependent manner. Based on RNA-sequencing (RNA-seq) of shoots and roots, we further identified 700 genes that were only differentially expressed in wild-type, not *sunn*, comparing AMF-colonized with non-colonized tissues.

## Materials and methods

### Plant material and plant growth

Experiments were performed using *M. truncatula* (Gaertn.), Jemalong A17. Seeds from *rdn1* (Jemalong J5) and *sunn-4* (Jemalong A17) were kindly provided by Professor Julia Frugoli (Schnabel *et al.*, 2005, 2011). The *mtcle53* mutant (NF10070) was obtained from the *Tnt1* insertion mutant collection at the Noble Research Institute (ecotype R108). Genotyping primers are listed in Supplementary Table S1 at *JXB* online. The cDNA used for expression analysis in the *mtpt4* mutant line (Fig. 3B, C) was provided by Professor Iver Jakobsen. Root length colonization data of the wild type (A17) and *mtpt4* in Supplementary Fig. S3C were derived from Watts-Williams *et al.* (2015). The *mtpt4* mutant line *mtpt4-1* was originally provided by Professor Maria Harrison.

Seed scarification was performed for 5–7 min in concentrated sulfuric acid (H_2_SO_4_) followed by five washes in cold Milli-Q water. Next, seeds were sterilized in 30% (v/v) chloride for 10 min followed by five washes with Milli-Q water. Seeds were imbibed in water for 1 h in the fridge and subsequently placed on wet filter paper in Petri dishes and vernalized at 5 °C for 48 h. Germination was initiated at room temperature in the dark for 24 h, whereafter seedlings were placed in the growth chamber until cotyledons were fully developed and green. Seedlings for hairy root transformation were prepared as described above, but were kept longer in the fridge (4–5 d) until primary roots had emerged. After transformation and recovery on solid agar, composite plants were grown as described below.

Plant growth experiments were performed according to the following procedure. Seedlings were planted in Cone-tainers (SC10, volume=164 ml) and placed in a growth chamber with 16 h days (22 °C) and 8 h nights (20 °C), 80% relative humidity, and a photon flux of 200 μmol m^−2^ s^−1^. The growth mixture consisted of 90% (v/v) sand mix [two parts coarse sand (0.4–0.8 mm) and one part fine sand (<0.4 mm)] and 10% (v/v) sieved soil (<5 mm). The soil came from a long-term P depletion field trial at University of Copenhagen and was γ-irradiated prior to preparing mycorrhizal inoculants (*Rhizophagus irregularis*, BEG87). For AMF experiments, plants were watered with 15 ml of low P solution every second day (Supplementary Table S2). Plants were harvested 21 days post-inoculation (dpi), except for the time-course experiment that was harvested at 14, 21, 28 and 35 dpi.

Data presented in Fig. 3A and Supplementary Fig. S3D–F came from plants grown in the following way. Seedlings were planted in Cone-tainers (SC10, volume=164 ml) and placed in a growth chamber with 16 h days (24 °C) and 8 h nights (22 °C), 60% relative humidity, and a photon flux of 450 μmol m^−2^ s^−1^. The growth mixture consisted of one part coarse sand (0.4–0.8 mm) and three parts fine sand (<0.4 mm). For AMF colonization with *R. irregularis* (DAOM 197198), 500 spores (Agronutrition, Toulouse, France) were placed 2 cm below the surface, whereafter seedlings were planted. Plants were watered with 15 ml of high P solution or low P solution (Supplementary Table S2) every second day for 2 weeks. During the last week, plants were watered daily with 10 ml of the respective nutrient solutions. Plants were harvested 19 dpi. Plants for the P resupply experiment (Supplementary Fig. S3D–F) were either kept on low P or shifted to high P (10 ml twice a day) and harvested after treatments for 48 h and 72 h.

Samples for the split-root experiments presented in Fig. 3B, C and Supplementary Fig. S3C came from a previously published experiment (Watts-Williams *et al.*, 2015). In brief, *M. truncatula* wild-type and *mtpt4* seedlings were planted in pots containing 50% semi-sterile soil and 50% sand (0.4–0.8 mm). After 16 d, the plants were transferred to a split-root system, which consisted of two adjoining pots with or without AMF inoculum, respectively. The root system of each plant was divided into two halves of similar size and planted in either of the two pots in the split-root system. The non-mycorrhizal (NM) pot contained 50% semi-sterile soil and 50% sand (0.4–0.8 mm), while the mycorrhizal (AMF) pot contained 10% soil inoculum (*R. irregularis*, BEG87), 40% semi-sterile soil. and 50% sand (0.4–0.8 mm). The soil–sand mix was supplemented with 20 mg P kg^−1^. Plants were harvested 21 d after transfer to the split-root system, and the expression level of *MtCLE53* was analyzed in the NM and AMF root halves of the same plant.

### Root staining and quantification of mycorrhiza colonization

**Fig. 1.**
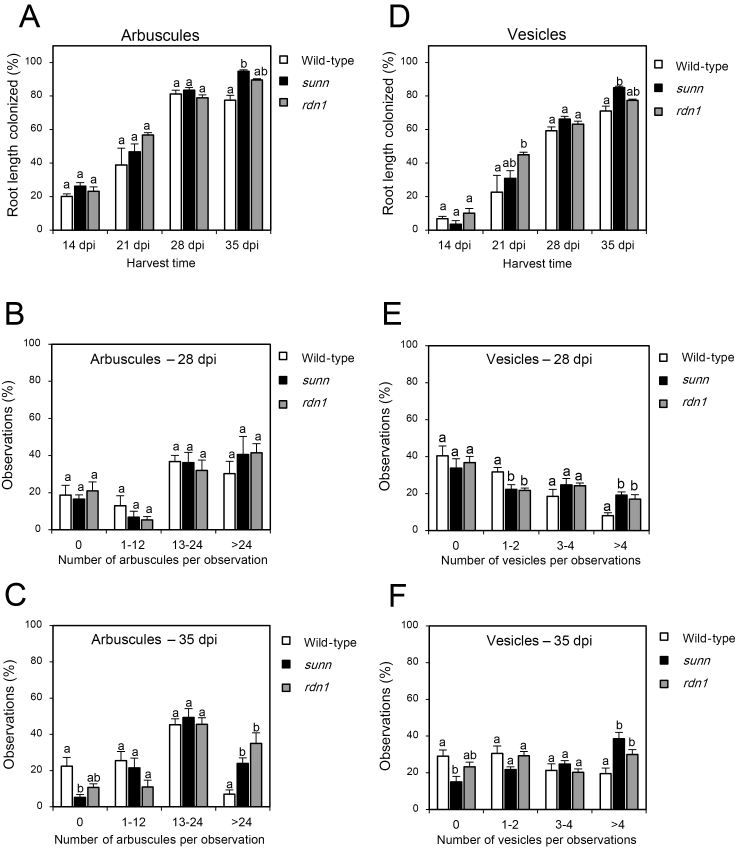
Development of arbuscular mycorrhizal colonization in the wild type, *sunn*, and *rdn1* over time. Root length colonization (%) of arbuscules (A) and vesicles (D) in the wild type, *sunn*, and *rdn1* at four different time points in days post-inoculation (dpi) based on McGonigle *et al.* (1990). The numbers of arbuscules (B, C) and vesicles (E, F) were counted within a pre-defined square of 0.08 mm^2^. To extract differences in arbuscule and vesicle intensities, observations were allocated to four groups based on the number of arbuscules (0, 1–12, 13–24, >24) or vesicles (0, 1–2, 3–4, >4) counted per observation. Observations per group are presented as a percentage (%). Observations were made in 100 individual root segments in each of four biological replicates per genotype. Bars are average values ±SEs. Statistically significant differences between averages within each group are labeled with a different letter based on one-way ANOVA followed by Tukey´s post-hoc test (*P*<0.05).

Rinsed root samples were stored in 50% ethanol. For visualization of fungal structures, roots were cleared in 10% KOH (w/v) at 90 °C for 10–15 min and stained in 0.05% (w/v) Trypan blue in lactoglycerol [H_2_O:glycerol:lactic acid (80%)=1:1:14] for 5 min. Stained roots were cut into 1 cm root segments and placed on microscope slides. AMF colonization was estimated using the magnified intersections method (McGonigle *et al.*, 1990) recording a minimum of 100 intersections per plant. In addition, the actual number of arbuscules and vesicles was counted within a pre-defined square of 0.08 mm^2^ at each intersection, using the horizontal cross-hair as the upper boundary. These measures are herein referred to as arbuscule and vesicle intensities and were used to prepare Fig. 1B, C, E, F and Supplementary Fig. S2.

### Golden gate cloning

The *MtCLE53* (Medtr8g463700.1) and *MtCLE53ΔPro4,7* coding sequences were obtained as synthetic DNA (TWIST Bioscience) with *Bsa*I restriction sites and overhangs compatible with the MoClo modular cloning system (Supplementary Table S3) (Engler *et al.*, 2008). Additional modular parts were obtained from the MoClo Tool Kit (Kit # 1000000044) (Weber *et al.*, 2011) and MoClo Plant Parts Kit (Kit # 1000000047) (Engler *et al.*, 2014). Prior to cloning, all acceptor vectors (1 g) were linearized with the appropriate enzyme (10 U) for 20 min at 37 °C in 50 μl of CutSmart buffer (1×) (NEB). Subsequently, samples were incubated with 10 U of rSAP (NEB) for an additional 20 min at 37 °C. The reaction mixture for Level 1 assembly included 0.5 μl of T4 ligase (400 U μl^–1^) (NEB), 1.5 μl of T4 buffer (10×), 0.5 μl of *Bsa*I-HFv2 (10 U μl^–1^) (NEB), 1.5 μl of BSA (1 μg μl^–1^) (NEB), dephosphorylated acceptor vector (100–200 ng), and Level 0 modules in 2:1 molar ratios of insert:acceptor. Sterile milli-Q water was added to 15 μl. The restriction–ligation program was 37 °C for 30 s, then 25 cycles of 37 °C for 3 min and 16 °C for 4 min, followed by 50 °C for 5 min, and 80 °C for 5 min. Assembled Level 1 modules were transformed into *Escherichia coli* and positive clones were selected on LB plates containing X-Gal (20 ng ml^–1^), isopropyl- β-d-thiogalactopyranoside (IPTG; 1 mM), and carbenicillin (100 μg ml^–1^). Assembly of Level 2 vectors was performed according to the above procedure using *Bbs*I-HF (NEB) instead of *Bsa*I. Selection was performed with kanamycin. Plasmids and final constructs are listed in Supplementary Table S3. Constructs were sequenced and transformed into *Agrobacterium rhizogenes* (strain Arqua1).

### Hairy root transformation

Seeds were scarified and sterilized as above. Sterilized seeds were placed in Petri dishes on solid Fahraeus medium (Supplementary Table S2), inverted, wrapped in tin foil, and placed in the fridge at 4 °C until primary roots had emerged. Primary root tips were excised 0.5 cm from the tip and dipped in PCR tubes containing 50 μl of high-density liquid culture of *A. rhizogenes* for 30 min. High-density liquid cultures appeared milky-white and were prepared from overnight cultures of *A. rhizogenes* grown in liquid LB. Bacteria from 50 ml of bacterial broth were gently pelleted, the supernatant removed, and bacteria were resuspended in water to a total volume of 250 μl. High-density cultures were either used directly for transformation with Level 2 constructs or mixed in equal volumes prior to dual transformation with Level 1 constructs 35S::DsRed and p35S::CLE53ΔPro4,7. After transformation, seedlings were placed on solid Fahraeus medium and covered with moist filter paper. Plates were placed vertically in a growth chamber with 16 h days (22 °C) and 8 h nights (20 °C), 80% relative humidity, and a photon flux of 200 μmol m^−2^ s^−1^. After 7 d, plants were transferred to pots and grown as described above. Expression of DsRed in transformed roots was observed using a fluorescence stereomicroscope (Leica M205).

### Phylogenetic analysis

A phylogenetic tree was generated based on multiple sequence alignment of all *M. truncatula* and *L. japonicus* CLE pre-proprotein sequences (Hastwell *et al.*, 2017) using ClustalX v2.1 (Larkin *et al.*, 2007). A Neighbor–Joining tree was constructed using 1000 bootstrap replications and subsequently modified using FigTree v1.4.3 (http://tree.bio.ed.ac.uk/software/figtree/).

### DNA extraction and PCR

Single leaves from individual mutant lines were ground and mixed with 100 μl of extraction buffer [200 mM Tris at pH 7.5, 250 mM NaCl, 75 mM EDTA, 0.5% (w/v) SDS] and centrifuged (12 000 *g*) for 1 min. Equal volumes of supernatant and isopropanol were mixed and centrifuged (12 000 *g*) for 10 min. The pellet was washed twice with 200 μl of 70% ethanol and dried before dissolving the DNA in 50 μl of water. The PCR mix comprised 1 μl of template DNA, 0.1 μl of GoTAG polymerase (5 U μl^–1^), 0.4 μl of dNTP mix (2.5 μM each), 0.8 μl of primer (forward and reverse, 10 μM each) (Supplementary Table S1), 4 μl of GoTAG reaction buffer, and Milli-Q water up to a final volume of 20 μl. The PCR program comprised denaturation at 95 °C for 2 min, amplification (95 °C for 40 s, 60 °C for 40 s, 72 °C for 1 min) for 30 cycles, and extension at 72 °C for 10 min.

### RNA extraction and reverse transcription quantitative–PCR (RT–qPCR)

Approximately 100 mg of tissue was mixed with 1 ml of TRI Reagent (Sigma-Aldrich) and incubated for 5 min at room temperature. Samples were centrifuged at 12 000 *g* for 10 min at 4 °C and the supernatant was mixed with 200 μl of chloroform, vortexed for 10 s, and incubated for 3 min at room temperature. The supernatant was mixed with a 0.5 vol. of isopropanol and incubated on ice for 1 h. RNA was pelleted by centrifugation (12 000 *g*) for 10 min and washed twice with 1 ml of 70% ethanol. Dried pellets were dissolved in 50 μl of diethyl pyrocarbonate (DEPC)-treated water. DNase treatment was performed with the TURBO DNA-free kit (Ambion) following the manufacturer’s instructions. For RT–qPCR, RNA yield and quality were assessed by gel electrophoresis and spectrophotometry (Nanodrop 2000, *A*_260_/*A*_280_>2.0).

Reverse transcription was performed by mixing 1 µg of RNA with 2 µl of oligo(dT)_23_ (50 µM), 1 µl of dNTP (2.5 μM each), and nuclease-free water up to 10 µl. Samples were heated to 65 °C for 5 min and placed on ice, and mixed with 2 µl of 10× M-MuLV buffer (NEB), 0.5 µl of M-MuLV reverse transcriptase (200 U µl^–1^) (NEB), 0.2 µl of RNase inhibitor, Murine (NEB), and 6.8 µl of nuclease-free water. Samples were incubated at 42 °C for 1 h followed by 65 °C for 20 min and 2 min on ice. All cDNA samples were diluted 4- to 8-fold. Primer sequences can be found in Supplementary Table S1. RT–qPCRs were performed in 10 µl using qPCR ready-mix 5× HOT FIREPol EvaGreen (Solis Biodyne) with final primer concentrations of 250 nM each. The thermal cycling (LightCycler 96, Roche) protocol was 95 °C for 15 min, followed by 45 cycles of 95 °C for 15 s, 58 °C for 20 s, and 72 °C for 20 s. A dissociation step was added to assess primer specificity. Differential gene expression was quantified based on the ΔΔCt method using MtUBQ as the reference gene. The number of biological replicates, the tissue used, and plant age at harvest are specified in the respective figure legends.

### Shoot phosphate measurements

Shoots were frozen in liquid nitrogen (N_2_) and ground with a pestle. Approximately 100 mg of ground shoot material and 1 ml of water were frozen and thawed three times, whereafter the supernatant was diluted 10 times. Pi was measured using a modified Malachite green assay (Kanno *et al.*, 2016): 140 μl of diluted supernatant was incubated with 30 μl of Reagent 1 [17.55 g (NH_4_)_6_Mo_7_O_24_·4 H_2_O per 1 liter of 3 M H_2_SO_4_) for 10 min. A 30 μl aliquot of Reagent 2 (3.5 g of polyvinyl alcohol and 0.35 g of Malachite green per 1 liter of water) was added and the absorbance at 600 nm was measured after 2 h.

### RNA-seq library preparation, transcriptome sequencing, and analysis

RNA was extracted from three biological replicates per sample type and purified with the RNeasy MiniElute Cleanup Kit (Qiagen) following the manufacturer’s instructions. Quality control was performed using a Bioanalyzer 2100 (Agilent). Library preparation was performed by GATC Biotech (Konstanz, Germany) using random primers, and samples were sequenced with an Illumina HiSeq 2500 (50 bp single reads, 30 million reads per sample). RNA-seq data sets were mapped to a re-annotated version of the *Medicago* genome (de Bang *et al.*, 2017*b*) to estimate raw counts and effective read lengths using Salmon (Patro *et al.*, 2014). Raw counts were normalized by median normalization, and differentially expression genes (DEGs) were estimated using the DEseq2 module from the Bioconductor R package (Love *et al.*, 2014). Gene expression was quantified as fragments per kilobase of transcript per million mapped reads (FPKM), and identified DEGs were required to have an adjusted *P*-value of <0.05. All data were analyzed at the MycPEP Gene Expression Atlas: http://bioinfo.noble.org/mycpep/. The MycPEP Gene Expression Atlas was implemented for *M. truncatula* based on the GEAUniversal system (Dai *et al.*, 2018) with a user-friendly interface, and with different analyses included: Gene Expression, Differential Expression, Co-expression, GO enrichment, and KEGG enrichment pathway. All RNA-seq data sets can be retrieved from the NCBI SRA database at https://www.ncbi.nlm.nih.gov/sra with the accession number PRJNA590945.

## Results

### AMF colonization is more intense in the hypernodulation mutants *sunn* and *rdn1*

The wild type and the hypernodulation mutants *sunn* and *rdn1* were inoculated with AMF and harvested at four different time points (14, 21, 28, and 35 dpi). Root length colonization and arbuscule and vesicle intensities were quantified in the colonized root systems at each time point (Fig. 1; Supplementary Fig. S1), and accompanied by gene expression analysis of AMF marker genes (Supplementary Fig. S1A, B). Root length colonization was quite similar at 14, 21, and 28 dpi, but significantly higher colonization of both arbuscules and vesicles was observed in *sunn* at 35 dpi compared with the wild type (Fig. 1A, D). For each observation, the number of arbuscules and vesicles was counted within a defined area of the root (0.08 mm^2^), in order to obtain a quantitative measure for colonization intensity. The average numbers of vesicles were significantly higher in *sunn* and *rdn1* at 28 dpi, and in *sunn* at 35 dpi, compared with the wild type (Supplementary Fig. S2A). The average numbers of arbuscules were similar among the genotypes at 21 dpi and 28 dpi, but significantly higher in *rdn1* than in the wild type at 35 dpi (Supplementary Fig. S2B). Grouping the number of arbuscules counted into a histogram revealed that observations with >24 arbuscules were more frequent (*P*<0.05) in both *sunn* and *rdn1* at 35 dpi compared with the wild type (Fig. 1C). Vesicle intensities were significantly higher in *sunn* and *rdn1* compared with the wild type at both 28 dpi and 35 dpi, where observations with >4 vesicles were more frequent (*P*<0.05) (Fig. 1E, F). Taken together, at later stages of the AMF symbiosis, significantly more vesicles (28 dpi and 35 dpi) and arbuscules (35 dpi) were present in *sunn* and *rdn1* roots compared with the wild type.

### MtCLE53 expression is specifically and independently induced by AMF colonization and phosphorus

**Fig. 2.**
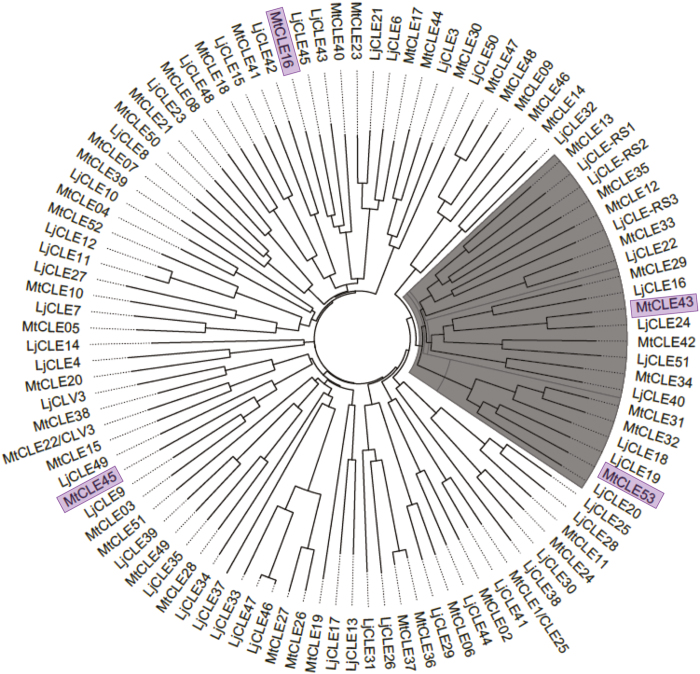
Phylogenetic analysis of *Lotus japonicus* and *Medicago truncatula* CLE pre-propeptide sequences. Unrooted phylogenetic tree based on all *L. japonicus* and *M. truncatula* CLEs. Pre-propeptide CLE sequences were aligned and a Neighbor–Joining tree with 1000 bootstrap replicates was generated in ClustalX v2.1. The polar tree layout was generated in FigTree v1.4.3. The identified clade is marked with gray. The four *CLE* genes induced by mycorrhiza in the RNA-sequencing data are highlighted.

To identify a potential CLE ligand for SUNN acting in AOM, expression of the entire *M. truncatula CLE* gene family (Hastwell *et al.*, 2017) was analyzed based on RNA-seq from AMF-colonized wild-type roots (21 dpi). Expression of four *CLE* genes was significantly induced in AMF-colonized roots compared with NM roots (adjusted *P*-value <0.05): *MtCLE16*, *MtCLE43*, *MtCLE45*, and *MtCLE53* (Supplementary Table S4). A phylogenetic analysis, based on full-length protein sequences of all CLEs from *M. truncatula* and *L. japonicus*, identified a clade containing the AMF-induced peptides MtCLE43 and MtCLE53 from *M. truncatula* and LjCLE19, LjCLE20, and LjCLE24 from *L. japonicus* (Handa *et al.*, 2015). In addition, the well-known AON CLEs MtCLE12, MtCLE13, LjCLE-RS1, LjCLE-RS2, and LjCLE-RS3, as well as the P-induced MtCLE33 (Müller *et al.*, 2019) were present in the clade (Fig. 2).

**Fig. 3.**
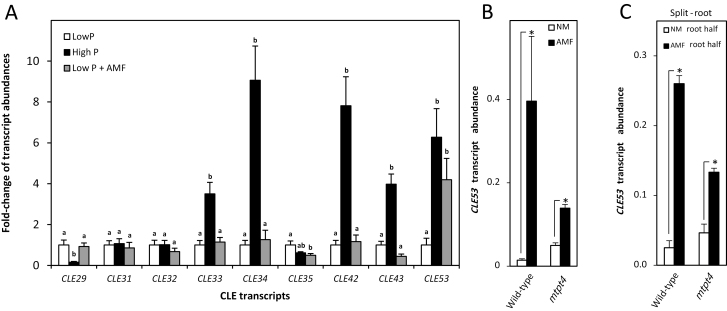
*CLE* transcript abundances measured by RT–qPCR. (A) Transcript abundance of *M. truncatula CLE* genes from the identified clade, except for *MtCLE12* and *MtCLE13*, in roots of plants grown at low P (6 μM Pi), high P (600 μM Pi), and low P inoculated with arbuscular mycorrhizal fungi (AMF) (6 μM Pi). For each transcript, low P expression levels were normalized to 1. Bars are average values ±SD (*n*=5). Different letters indicate significant differences between individual *CLE* transcript abundances based on one-way ANOVA followed by Tukey´s post-hoc test (*P*<0.05). (B) Transcriptional response of *MtCLE53* to AMF colonization in the wild type and *mtpt4*. (C) Transcriptional responses in the wild type and *mtpt4* of *MtCLE53* in the AMF-colonized root half (AMF) compared with the non-colonized root half (NM) in a split-root system. Bars are averages ± SD (*n*=3). Significant differences in (B) and (C) are marked with asterisks (*) and were calculated based on two-sided Student’s *t*-test (*P*<0.05).

Transcriptional responses to P levels and early stages of AMF colonization of the *CLE* genes present in the clade were analyzed, except for the nodule-specific *MtCLE12* and *MtCLE13*, as their transcription is not affected by AMF colonization (Müller *et al.*, 2019). Expression levels were determined in roots from plants grown at high P conditions (600 μM Pi) without AMF and low P conditions (6 μM Pi) with and without AMF (Fig. 3A). AMF colonization was at an early stage with a root length colonization of 20%. In comparison with low P, *MtCLE53* expression was the only gene significantly induced by both high P and AMF among the analyzed *CLE* transcripts (Fig. 3A). In addition, transcript levels of *MtCLE33*, *MtCLE34*, *MtCLE42*, and *MtCLE43* significantly increased in response to the high P treatment compared with the low P treatment, while *MtCLE29* transcription decreased (Fig. 3A). Importantly, AMF-colonized plants in this experiment remained P deficient, as shown by similar expression of the P deficiency marker gene *PHOSPHORUS STARVATION INDUCED* (*MtPSI*) and similar shoot Pi contents between the low P and AMF treatments (Supplementary Fig. S3A, B). This underlined that *MtCLE53* induction at low P was dependent on AMF and not caused by an improved P status. Further supporting this, *MtCLE53* expression significantly increased by AMF colonization in the Pi transporter mutant *mtpt4*, which is impaired in AMF-derived Pi uptake (Javot *et al.*, 2007) (Fig. 3B). *MtCLE53* induction by AMF colonization in *mtpt4* was less pronounced compared with the wild type; however, this might be a reflection of the much reduced AMF colonization in *mtpt4* (~50% compared with the wild type) (Supplementary Fig. S3C).

*MtCLE53* is specifically expressed in roots (de Bang *et al.*, 2017*b*) and we thus analyzed *MtCLE53* expression in a split-root set-up, as a local increase in expression would support AMF-dependent induction of *MtCLE53* and a potential role in AOM. Indeed, *MtCLE53* expression significantly increased in AMF-colonized root halves compared with NM root halves, showing that AMF-dependent *MtCLE53* induction was a local response (Fig. 3C). Since *MtCLE53* expression was high in P-sufficient plants (Fig. 3A), the short-term response to Pi was tested by resupplying Pi to plants grown under low P conditions. Shoot Pi content and expression of *MtPSI* in Pi-resupplied plants reached high P levels within 48 h, while there were no effects on *MtCLE53* expression in response to Pi resupply (Supplementary Fig. S3D–F). Taken together, the above results indicated a role for MtCLE53 in regulating susceptibility to AMF colonization based on both AMF colonization itself and longer term P availability..

### MtCLE53 and SUNN define a genetic module regulating AMF colonization

**Fig. 4.**
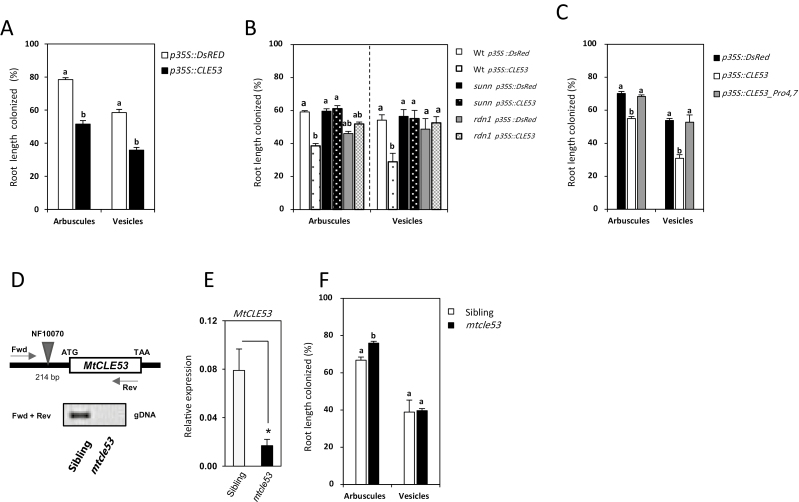
Root length colonization by mycorrhizal arbuscules and vesicles in different genetic backgrounds. (A) Composite plants overexpressing *p35S::CLE53* compared with empty vector controls (*p35S::DsRed*) in the wild-type background. (B) Composite plants overexpressing *p35S::CLE53* compared with empty vector controls (*p35S::DsRed*) in the wild-type, *sunn*, and *rdn1* backgrounds. (C) Composite wild-type plants overexpressing a native version of *MtCLE53* (*p35S::CLE53*) and a mutated version with prolines changed to glycines (*p35S::CLE53_Pro4,7*) compared with the empty vector control (*p35S::DsRed*). Root length colonization was assessed in individual root segments (*n*=100) in four biological replicates. Bars are averages ± SEs. Statistically significant differences between averages within each group are labeled with different letter based on one-way ANOVA followed by Tukey´s post-hoc test (*P*<0.05). (D) The *mtcle53* mutant line NF10070 has a *Tnt1* insert 214 bp upstream of the *MtCLE53* ATG start codon. Based on PCR on genomic DNA (gDNA) using *MtCLE53*-specific forward (fwd) and reverse (rev) primers, homozygous *mtcle53* mutants were identified in a segregating population of NF10070 siblings. (E) Relative expression levels of *MtCLE53* in *mtcle53* and heterozygous siblings based on qRT–PCR of AMF-colonized roots. Bars are averages ± SDs. Significant differences are marked with an asterisk as calculated by two-sided Student’s *t*-test (*P*<0.05). (F) Root length colonization by arbuscules and vesicles in *mtcle53* and siblings in analyzed root segments (*n*=160) of eight biological replicates. Bars are averages ± SEs. Statistically significant differences between averages within each group are labeled with a different letter based on one-way ANOVA followed by Tukey’s post-hoc test (*P*<0.05)

Composite plants constitutively overexpressing *MtCLE53* were prepared in the wild-type background using hairy root transformation (Supplementary Fig. S3G). *MtCLE53* overexpression significantly reduced arbuscule and vesicle root length colonization compared with roots overexpressing *DsRed* (Fig. 4A). *MtCLE53* overexpression in the wild-type, *sunn*, and *rdn1* genetic backgrounds confirmed the effect of MtCLE53 on AMF colonization, and showed that this effect was dependent on both SUNN and RDN1 (Fig. 4B). The plasmid used for transformation carried both a *p35S::CLE53* construct and a *p35S::DsRed* construct (Supplementary Table S3), enabling identification of *MtCLE53*-overexpressing roots using fluorescence microscopy. The plants analyzed had one or two transformed roots out of a total of 6–12 individual roots in the whole root system. Since AMF colonization was assessed in the entire root system and thus primarily in non-transformed roots, it can be hypothesized that MtCLE53 travels from root to shoot to activate SUNN, but this requires additional experiments for confirmation.

An *mtcle53* mutant line (NF10070) was identified by reverse genetics from the *Tnt1* mutant collection held at the Noble Research Institute (https://medicago-mutant.noble.org/mutant/) (Tadege *et al.*, 2008; Cheng *et al.*, 2014). The *Tnt1* insert was located in the promotor region 214 bp upstream of the start codon and segregated in a Mendelian fashion with ~25% homozygous individuals in a segregating population. Expression of *MtCLE53* in homozygous *mtcle53* mutants compared with heterozygous siblings was reduced by ~80%, but the insertion did not completely abolish *MtCLE53* transcription (Fig. 4D, E). The reduced *MtCLE53* transcription resulted in significantly (*P*<0.001) higher root length colonization by arbuscules in the homozygous *mtcle53* mutant (76%) compared with its heterozygous siblings (67%) at 21 dpi. No difference in root length colonization was observed for vesicles (Fig. 4F).

The missing effect of *MtCLE53* overexpression in *rdn1* (Fig. 4B) indicated that the MtCLE53 peptide may require hydroxyproline tri-arabinosylation to activate SUNN. To analyze this aspect in more detail, an *MtCLE53* coding sequence resulting in mutated prolines at positions four and seven (MtCLE53ΔPro4,7), probably hindering tri-arabinosylation of the peptide, was overexpressed in composite plants. The mutated MtCLE53 version had no effect on AMF colonization, which was similar to roots overexpressing *DsRed* (Fig. 4C). Taken together, our results indicate that MtCLE53 is produced locally in roots in response to AMF colonization or high P levels to negatively regulate AMF colonization. MtCLE53 acts upstream of or in parallel to MtPT4 and is dependent on SUNN and RDN1 to exert its function.

### Mycorrhiza affect transcriptional responses in a SUNN-dependent manner

**Fig. 5.**
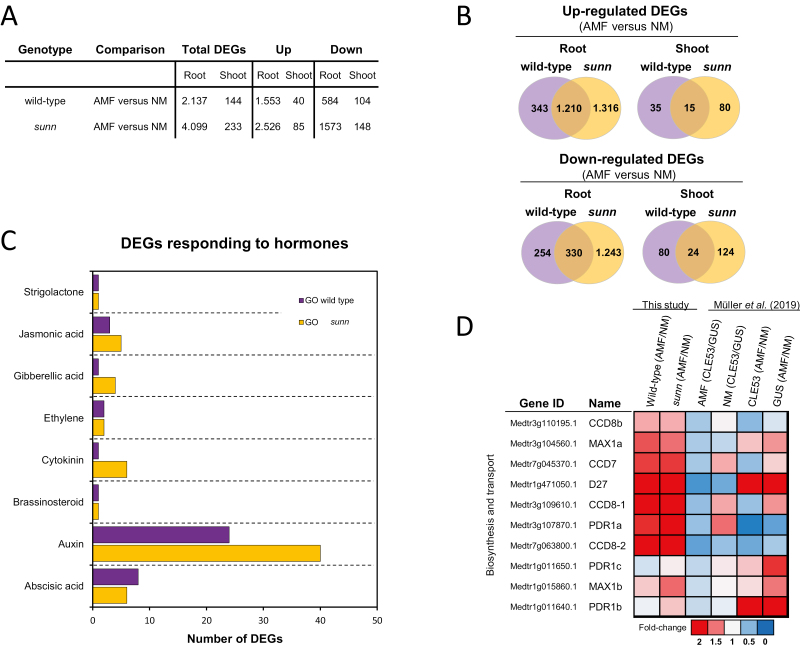
RNA-sequencing analysis of wild-type and *sunn* roots and shoots from plants colonized by arbuscular mycorrhizal fungi (AMF) or from plants not colonized by AMF (NM). (A) Differentially expressed genes (DEGs) in roots and shoots of the wild type and *sunn*. The total number of DEGs (AMF versus NM) and the numbers of up- and down-regulated DEGs are listed separately. Statistically significant differences between transcript levels were calculated using DESeq2 (adjusted *P-*value <0.05). (B) Venn diagrams comparing up- and down-regulated DEGs (AMF versus NM) in roots and shoot of AMF-colonized wild type and *sunn*, respectively. (C) Enriched GO terms related to the phytohormones auxin, abscisic acid, cytokinin, brassinosteroid, ethylene, gibberellic acid, jasmonic acid, and strigolactone. The analysis was based on DEGs in wild-type and *sunn* roots. (D) Transcriptional changes in strigolactone biosynthesis and transport gene expression levels compared between roots colonized by AMF or not (NM), either in the wild type and *sunn* (this study), or in MtCLE53- or GUS-overexpressing roots (Müller *et al.*, 2019). Transcriptional changes are represented as fold changes using a heat map.

RNA-seq was performed on non-colonized and AMF-colonized roots and shoots from the wild type and *sunn* at 21 dpi. Data can be accessed and analyzed at the MycPEP homepage (http://bioinfo.noble.org/mycpep/), which includes the possibility of performing custom differential expression analysis. Twenty AMF marker genes were all induced by AMF colonization in both wild-type and *sunn* roots (Supplementary Table S5). Large transcriptional changes were induced in AMF-colonized roots compared with non-colonized roots, with 2137 DEGs in the wild type and 4099 DEGs in *sunn* (adjusted *P-*value <0.05) (Fig. 5A, B; Supplementary Table S6). Transcriptional responses in shoots were much less pronounced with 144 DEGs in the wild type and 233 DEGs in *sunn* comparing shoots from non-colonized and AMF-colonized plants (adjusted *P*-value <0.05) (Fig. 5A, B; Supplementary Table S6).

A Kyoto Encyclopedia of Genes and Genomes (KEGG) pathway analysis and a Gene Ontology (GO) term enrichment analysis were performed via the MycPEP homepage. In total, 20 KEGG pathways were enriched (adjusted *P*-value <0.05), of which the most striking difference between the wild type and *sunn* was found in roots for DEGs belonging to the pathways ‘plant hormone signal transduction’ and ‘phenylpropanoid biosynthesis’ (Supplementary Fig. S4; Supplementary Table S7). A total of 32 and 49 GO terms were enriched in the wild type and *sunn*, respectively (Supplementary Table S8). GO terms related to ‘fatty acid metabolic processes’ (GO:0006631) and ‘transmembrane transport’ (GO:0055085) were exclusively enriched in the wild type (Supplementary Table S8), while ‘cell wall organization’ (GO:0071555) and ‘polysaccharide metabolic process’ (GO:0005976) were exclusive to *sunn* (Supplementary Table S8). In addition, a higher number of DEGs belonging to ‘response to auxin’ (GO:0009733) were enriched in *sunn* compared with the wild type (Fig. 5C; Supplementary Table S9).

Based on RNA-seq, Müller *et al.* (2019) showed that expression levels of the strigolactone biosynthesis genes *MtD27* and *MtMAX1a* were reduced in both AMF and NM roots overexpressing *MtCLE53* compared with controls overexpressing β *-glucuronidase* (*GUS*) (Fig. 5D; Supplementary Table S10). In contrast, AMF colonization increased the expression of *MtD27* and *MtMAX1a* compared with NM roots irrespective of *MtCLE53* overexpression (Fig. 5D). In the present study, AMF colonization also significantly increased transcription levels of *MtD27* and *MtMAX1a* compared with NM roots in both the wild type and *sunn* (Fig. 5D). We next asked which genes were up- and down-regulated by AMF in a SUNN-dependent manner in roots and shoots, respectively (i.e. DEGs only regulated in the wild type) (Fig. 5B). In roots, the most up-regulated SUNN-dependent gene was a cycloartenol synthase (sterol metabolism), while the most down-regulated DEG was an allene oxide synthase (jasmonic acid biosynthesis) (Supplementary Table S11). An NF-YA transcription factor (Medtr7g106450.1) was significantly down-regulated in both root and shoot in a SUNN-dependent manner, as also shown by Schaarschmidt *et al.* (2013) (Supplementary Table S11). Additional interesting DEGs up-regulated in a SUNN-dependent manner were a thioredoxin and a glutaredoxin-C1 protein of special interest as they also were significantly up-regulated by *MtCLE53* overexpression (Supplementary Table S11) (Müller *et al.*, 2019).

## Discussion

Symbiosis with AMF provides the plant with P and other nutrients in return for carbohydrates and lipids. To balance the costs associated with AMF symbiosis, plants regulate the interaction via the AOM mechanism that deploys long-distance root-to-shoot-to-root signaling to inhibit subsequent infection events (Vierheilig *et al.*, 2000; Meixner *et al.*, 2005). In this study, we show that the MtCLE53 peptide negatively regulates AMF colonization in a SUNN- and RDN1-dependent manner, and that *MtCLE53* expression is induced locally by AMF colonization itself and by P. As such, MtCLE53 is a candidate to be the long-sought root-to-shoot signal in AOM.

### CLE peptides regulating symbiosis belong to the same phylogenetic clade

The *M. truncatula* and *L. japonicus* genomes encode at total of 52 and 53 *CLE* genes, respectively, but their functions remain largely unknown (Hastwell *et al.*, 2017). Our phylogenetic analysis identified a clade composed of 11 *M. truncatula* and 11 *L. japonicus* CLEs that, among others, included the known AON CLEs (Fig. 2). The *M. truncatula* CLEs present in the identified clade corresponded to those of Group 3 in the study by Goad *et al.* (2017), who performed a clustering analysis of 1628 CLE pre-propeptide sequences obtained from 57 different plant genomes. In addition to known nodulation CLEs from the legumes *M. truncatula* and soybean, Group 3 also comprised CLEs from non-nodulating plants such as *Brachypodium distachyon*, rice, sorghum, and several brassicas, indicating that the functions of these CLEs were not restricted to nodulation (Goad *et al.*, 2017). Indeed, the clade identified in the present study included *L. japonicus* CLEs previously shown to respond to P and AMF colonization at the transcriptional level: *LjCLE19*, *LjCLE20*, and *LjCLE24* (Funayama-Noguchi *et al.*, 2011; Handa *et al.*, 2015). We further showed that *MtCLE29*, *MtCLE33*, *MtCLE34*, *MtCLE42*, *MtCLE43*, and *MtCLE53* all responded to P, while AMF colonization also induced *MtCLE53* expression (Fig. 3). This is in contrast to the study by Müller *et al.* (2019) who reported that *MtCLE53* only responded to AMF colonization. MtCLE53 is the putative functional ortholog of LjCLE19 and LjCLE20, and the expression pattern presented here is consistent with those reported for *LjCLE19* and *LjCLE20*. *MtCLE43* expression was induced by AMF colonization in the RNA-seq analysis (Supplementary Table S4), but this induction was not consistent between experiments (Fig. 3). *MtCLE43* expression was not analyzed by Müller *et al.* (2019), possibly because the *MtCLE43* gene was only recently annotated (Hastwell *et al.*, 2017). Since MtCLE43 is the putative functional ortholog of the AMF-induced LjCLE24, future studies should further investigate a possible role for MtCLE43 in AOM.

Some of the CLEs present in the identified clade (Fig. 2) have previously been reported to respond to nutrients other than P and to nodulation (de Bang *et al.*, 2017*b*). For example, *MtCLE34* expression was reduced in nitrogen-, P-, and sulfur-deficient roots, while *MtCLE53* expression was reduced in P- and sulfur-deficient roots. Furthermore, expression levels of both *MtCLE53* and *MtCLE33* were transiently induced 12 h post-inoculation with rhizobia, while *MtCLE29*, *MtCLE34*, *MtCLE35*, and *MtCLE42* were all induced in rhizobia-inoculated roots and at various stages of nodule development (de Bang *et al.*, 2017*b*). The expression of *CLE* genes belonging to Group 3 (Goad *et al.*, 2017) from species other than *M. truncatula* has also been shown to respond to nutrient status and AMF colonization. Müller *et al.* (2019) analyzed the expression pattern of *B. distachyon* Group 3 *CLE* genes and identified three *CLE* genes that responded to P and three *CLE* genes that responded to AMF colonization, of which one *CLE* transcript responded to both P and AMF. In *A. thaliana*, the Group 3 CLEs AtCLE1, AtCLE2, AtCLE3, and AtCLE4 modulated lateral root growth in response to either nitrogen or sulfur deficiencies (Araya *et al.*, 2014; Dong *et al.*, 2019). Hence, it appears that Group 3 CLEs may function to regulate symbiotic interactions and root growth in response to various nutrient deficiencies in a broader context. Since brassicas engage neither with rhizobia nor with AMF, it would be interesting to study the role of the CLE–SUNN module in other plant–microbe interactions.

### Is MtCLE53 a systemic and tri-arabinosylated peptide signal?

Both the AOM and AON mechanisms are based on systemic long-distance signaling that depends on shoot active receptors. The AON mechanism is activated by root-derived CLE peptides that ascend to the shoot, and in *M. truncatula*, *L. japonicus*, and soybean these CLEs are known to require tri-arabinosylation to function (Mortier *et al.*, 2010; Okamoto *et al.*, 2013; Imin *et al.*, 2018; Hastwell *et al.*, 2019). In *M. truncatula*, genetic evidence has shown that the HPAT called RDN1 is required for the function of MtCLE12, but not MtCLE13 (Kassaw *et al.*, 2017; Imin *et al.*, 2018). In this study, we showed that ectopic overexpression of *MtCLE53* in the *rdn1* genetic background did not reduce AMF root colonization in comparison with the empty vector control (Fig. 4B). Furthermore, overexpression of a modified version of *MtCLE53*, where the prolines in positions four and seven were modified to glycine, did not reduce AMF root length colonization in the wild-type background (Fig. 4C). In addition, greater intensities of arbuscules and vesicles were observed in AMF-colonized *rdn1* roots compared with the wild type (Fig. 1C, E, F), indicating that AOM was impaired in the *rdn1* mutant. Collectively, the above results strongly suggest that the MtCLE53 peptide requires tri-arabinosylation to induce AOM; however, this should be confirmed by structural analysis based on MS.

*MtCLE53* is specifically expressed in roots (de Bang *et al.*, 2017*b*) and was locally induced in the AMF-colonized root half compared with the non-mycorrhizal root half in a split-root system (Fig. 3C). Furthermore, Müller *et al.* (2019) reported that *MtCLE53* expression localized to the stele of AMF-colonized roots, and that ectopic overexpression of *MtCLE53* using the promotor of the cortex-specific *MtBCP1* gene had no effect on AMF colonization. This implied that MtCLE53 could not be transported from the cortex to the stele and that xylem proximity was required for MtCLE53 function (Müller *et al.*, 2019). Expression of *SUNN* localized to the vasculature of shoots, petioles, roots, and nodules, while *RDN1* expression was restricted to the root vasculature in hairy root transformed plants (Schnabel *et al*., 2011, 2012). Collectively, the expression of *MtCLE53*, *SUNN*, and *RDN1* is confined to the vasculature, and thus both a local and a systemic action can be envisaged. While most AON CLEs are known to act systemically, AON CLEs in soybean act both locally and systemically (Reid *et al.*, 2011*b*). Similarly, Mortier *et al.* (2010) reported a local effect of MtCLE12 and MtCLE13 on nodulation, though the local effect was less strong than the systemic effect. Whether MtCLE53 and MtCLE33 act systemically or locally remains to be elucidated, but in future studies this should be investigated through grafting and split-root experiments.

### SUNN-dependent signaling and downstream targets

In this study, we compared the transcriptional responses to AMF colonization in both the wild type and *sunn* (Fig. 5; Supplementary Table S6). KEGG pathway analysis and GO term analysis provided insight into possible functional categories enriched among the DEGs, which among others included the KEGG pathway ‘plant hormone signal transduction’ and the GO term ‘response to hormone’ (Supplementary Tables S7, S8). Müller *et al.* (2019) identified strigolactone biosynthesis to be a downstream target for AOM. However, we did not find any differences in the expression of strigolactone biosynthesis genes when comparing AMF-colonized roots of the wild type and *sunn* (Fig. 5D; Supplementary Table S10). Another possible SUNN-dependent link to strigolactones was found in the shoot, where the transcript of a sesquiterpene synthase was down-regulated in the wild type, but not in *sunn* (Supplementary Table S11). This is interesting, as sesquiterpenes constitute a group of secondary metabolites that include strigolactones, but further studies are required to confirm any role for this gene in AOM. Another gene important for hormone biosynthesis, an allene oxide synthase that catalyzes the first committed step in jasmonic acid (JA) biosynthesis, was found to be down-regulated in AMF-colonized wild-type roots, but not in AMF-colonized *sunn* roots (Supplementary Table S11). This is congruent with previous studies in soybean, where the expression of JA biosynthesis genes and JA response genes was found to be NARK (nodule autoregulation receptor kinase) dependent in rhizobia-inoculated plants (Kinkema and Gresshoff 2008).

Co-inoculation with AMF and rhizobia resulted in an NARK-dependent inhibition of AMF colonization in soybean (Sakamoto *et al.*, 2013), while pre-inoculation with either rhizobia or AMF resulted in reduced AMF colonization and nodulation in alfalfa split-root systems, respectively (Catford *et al.*, 2003). This suggests that AON and AOM share not only shoot-active SUNN as a regulatory node, but possibly also part of the downstream pathway. It could be speculated that nodulation-specific and AMF-specific CLE peptides are able to impair colonization of both symbionts, and vice versa. Müller *et al.* (2019) showed that overexpression of *MtCLE53* reduced the expression of several strigolactone biosynthesis genes including the β-carotene isomerase MtD27, the first enzyme in the strigolactone biosynthesis pathway. They further demonstrated that overexpression of both *MtCLE33* and *MtCLE53* reduced *MtD27* expression in a SUNN-dependent manner. However, overexpression of *MtCLE13* did not reduce *MtD27* expression in AMF-colonized roots and did not affect AMF colonization compared with the *GUS* control (Müller *et al.*, 2019). In contrast, Gautrat *et al.* (2019) reported a down-regulation of *MtD27* by *MtCLE13* overexpression in non-inoculated roots. In the present study, *MtD27* expression increased in response to AMF colonization in both the wild type and *sunn* (Fig. 5D), which was also observed in AMF-colonized roots overexpressing *MtCLE53* compared with non-mycorrhizal roots overexpressing *MtCLE53* (Fig. 5D; Müller *et al.*, 2019). Overexpression of *MtCLE33* and *MtCLE53* reduced strigolactone levels in non-inoculated roots in a SUNN-dependent manner; however, no significant differences in strigolactone levels were measured between the wild type and *sunn* in roots overexpressing *GUS* (Müller *et al.*, 2019). Similarly, no differences in strigolactone levels were measured in non-inoculated roots of pea *nark* and *clv2* mutants compared with the wild type, while strigolactone levels increased in the pea *rdn1* mutant compared with the wild type (Foo *et al.*, 2014). Collectively, further studies are required to confirm an effect on strigolactone biosynthesis directly downstream of SUNN, as it appears that this regulation may involve other as yet unknown factors.

**Fig. 6.**
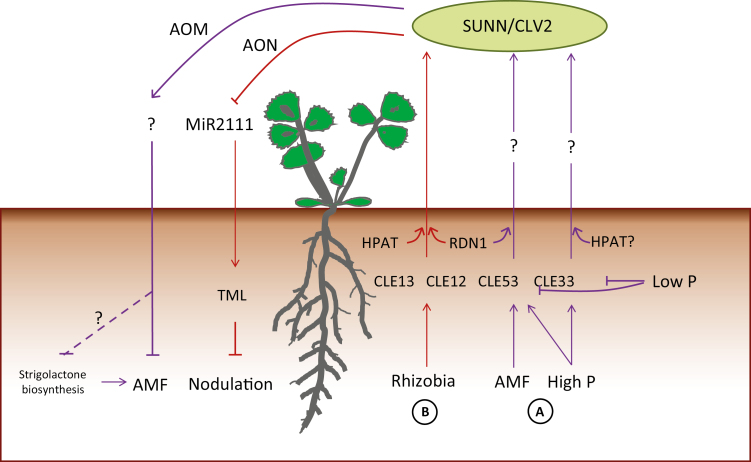
Proposed model for autoregulation of mycorrhization (AOM) (A) compared with autoregulation of nodulation (B). (A) Arbuscular mycorrhizal fungi (AMF) and high phosphorus (P) induce expression of *MtCLE53* and *MtCLE33* compared with low P expression levels. *MtCLE53* and *MtCLE33* are only expressed in roots. The mature MtCLE53 and MtCLE33 peptides could be root–shoot ascending signals initiating the systemic AOM mechanisms. AOM is dependent on the leucine-rich repeat receptor-like kinase SUPER NUMERIC NODULES (SUNN) and CLAVATA2 (CLV2). MtCLE53 is further dependent on the hydroxyproline *O*-arabinosyl transferase (HPAT) enzyme ROOT-DETERMINED NODULATION 1 (RDN1), while the requirement for post-translational modification of MtCLE33 is unknown. Signaling events downstream of SUNN possibly reduce strigolactone biosynthesis. (B) Rhizobia induce MtCLE12 and MtCLE13 in infection sites and developing nodules. Both peptides require tri-arabinose modification, MtCLE12 by RDN1 and MtCLE13 by an unknown HPAT. The mature MtCLE12 and MtCLE13 peptides ascend to the shoot to activate SUNN. SUNN signaling represses the shoot-to-root mobile miRNA miR2111, which in turn releases expression of the Kelch-repeat F-box protein TOO MUCH LOVE (TML). TML inhibits further nodulation.

In conclusion, we identified a genetic module consisting of MtCLE53, SUNN, and RDN1 that negatively regulates AMF colonization. This work extends the recent findings of Müller *et al.* (2019) by demonstrating *MtCLE53* induction by P, as well as a role for RDN1 in regulating AMF colonization. We propose a model in which root-derived tri-arabinosylated CLE peptides are produced in roots in response to AMF colonization and P (Fig. 6). The RNA-seq data of AMF-colonized wild-type and *sunn* plants provides a valuable resource for future identification of additional AOM genes.

## Supplementary data

Supplementary data are available at *JXB* online.

Fig. S1. Marker gene expression and pictures of AMF-colonized roots.

Fig. S2. Arbuscule and vesicle intensities.

Fig. S3. Gene expression analyses and phosphate contents.

Fig. S4. KEGG pathway analysis.

Table S1. Primers used in this study.

Table S2. Nutrient solution recipes.

Table S3. Plasmids and constructs used for GoldenGate cloning.

Table S4. *CLE* gene expression levels in AMF-colonized wild type and *sunn.*

Table S5. Marker gene expression in AMF-colonized wild type and *sunn.*

Table S6. Differentially expressed genes in RNA-sequencing analysis.

Table S7. KEGG pathway analysis.

Table S8. GO term enrichment analysis.

Table S9. Differentially expressed genes associated with hormone GO terms.

Table S10. Transcriptional changes in strigolactone genes.

Table S11. SUNN-dependent transcriptional responses.

eraa193_suppl_Supplementary_Figures

eraa193_suppl_Supplementary_Tables
