# Supplementary material for: The CLE53–SUNN genetic pathway negatively regulates arbuscular mycorrhiza root colonization in Medicago truncatula
Source: J Exp Bot. 2020 Apr 20;71(16):4972–84. doi: 10.1093/jxb/eraa193 (PMC7410177; doi:10.1093/jxb/eraa193)
Supplement: eraa193_suppl_Supplementary_Figures [file eraa193_suppl_supplementary_figures.pdf]

Supplementary figure legends

Fig. S1. (A,B) Transcript abundances of the AMF marker genes *MtPT4* (plant) and *α-TUBULIN* (fungus) in wild-type and *sunn*. Marks are averages ± standard errors (n=3). There were no significant differences in transcript levels between wild-type and *sunn* at any time point based on two-way ANOVA ($p < 0.05$). (C) Representative pictures of colonized roots of wild-type, *sunn* and *rdn1* at the four different time-points (days post inoculation (dpi)).

Fig. S2. Arbuscule and vesicle intensity in colonized roots of wild-type, *sunn* and *rdn1*. Number of vesicles (A) and arbuscules (B) were counted within a predefined square of 0.08 mm^2 in individual root segments of four biological replicates in wild-type, *sunn* and *rdn1* at three different time points (days post inoculation (dpi)). 100 observations were made per replicate. Bars are averages + standard errors (n = 100). Different letters indicate significant differences within genotypes at each time point as calculated by one-way ANOVA followed by Tukey´s posthoc test ($p<0.05$).

Fig. S3. Gene expression analyses and phosphate contents. (A) Relative gene expression levels of *PHOSPHATE STARVATION INDUCED* (*MtPSI*) and *MtPT4* at sufficient phosphorus (High P), phosphorus deficiency (Low P) and Low P plants colonized with arbuscular mycorrhiza (AMF). Expression levels were analyzed with RT-qPCR using the delta-delta Ct method (n=4). (B) Shoot phosphate (Pi) content (mg $plant^{-1}$) measured using the Malachite-green assay (n = 3). (C) Total root length colonization by arbuscules in wild-type and *mtpt4* plants derived from (Watts-Williams *et al*., 2015) (n = 3). (D,E) Gene expression levels of *MtCLE53* and *MtPSI* in Low P roots re-supplied or not with Pi for 48 hours and 72 hours (n = 4). (F) Shoot Pi content (mg $plant^{-1}$) in Low P plants re-supplied with Pi or not for 48 hours and 72 hours (n = 4). (G) *MtCLE53* expression levels in composite plants overexpressing *p35S::CLE53*, *p35S::DsRed* or a mutated version of *MtCLE53* where the prolines in positions four and seven were modified to glycines *p35S::CLE53ΔPro4,7* (n = 4). Statistically significant differences were calculated based on one-way ANOVA followed by Tukey´s posthoc test ($p < 0.05$) and are marked with letters, while significant differences calculated based on two-sided Student´s t-test ($p < 0.05$) are marked with an asterisk (*). Non-significant differences are denoted "ns".

Fig. S4. KEGG pathway analysis. Enriched pathways were identified among all DEGs in roots of wild-type and *sunn*, and are marked with an X to indicate in which genotype they were enriched. Gene ratios, i.e. number of enriched DEGs divided by the total number of genes associated with a given pathway, were calculated for pathways enriched in at least one genotype. Circle sizes refer to the number of DEGs enriched in the individual pathways.

Figure S1

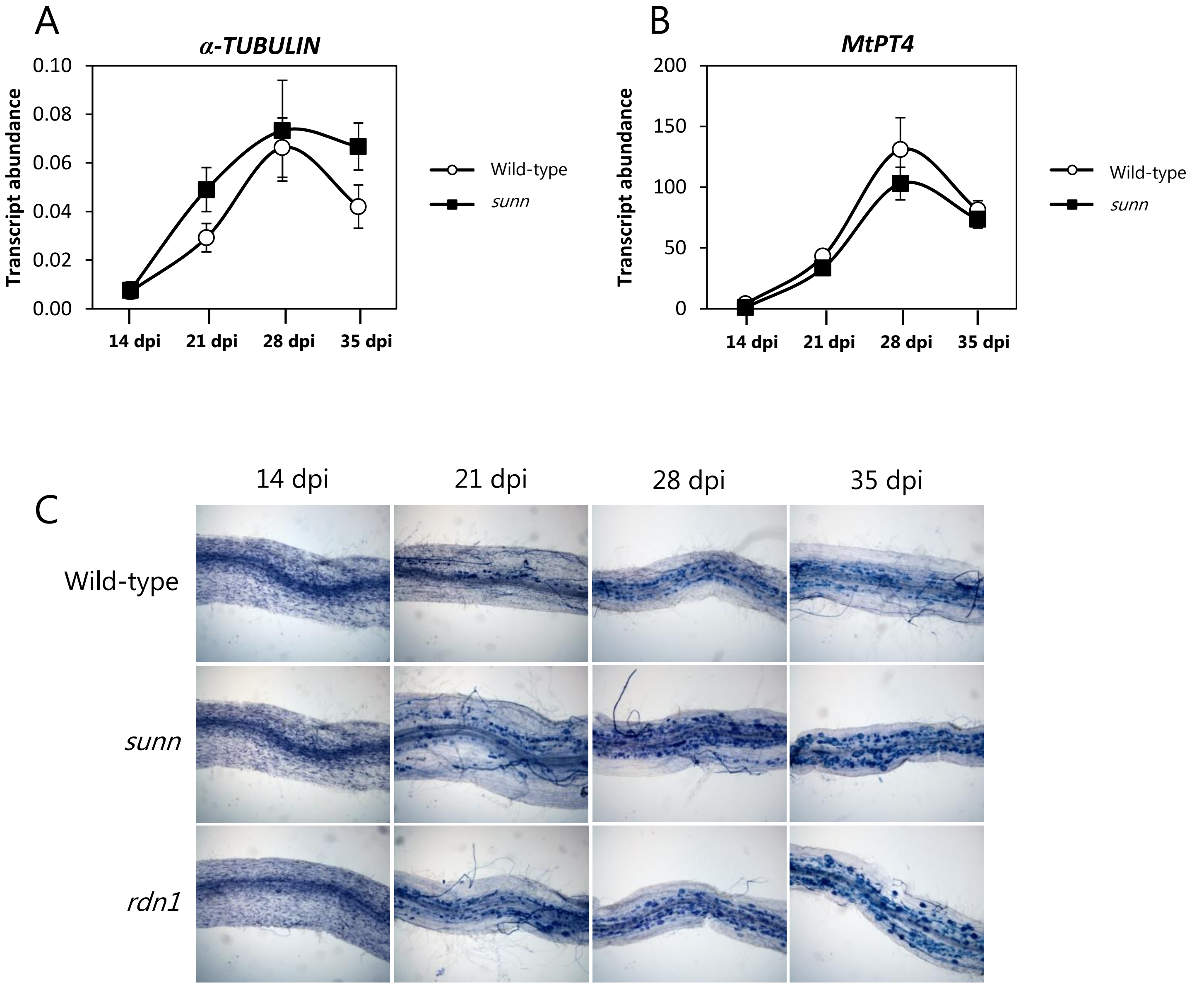

A
α-TUBULIN
Transcript abundance
0.10
0.08
0.06
0.04
0.02
0.00
14 dpi
21 dpi
28 dpi
35 dpi
Wild-type
sunn
B
MtPT4
Transcript abundance
200
150
100
50
0
14 dpi
21 dpi
28 dpi
35 dpi
Wild-type
sunn
C
14 dpi
21 dpi
28 dpi
35 dpi
Wild-type
sunn
rdn1

Figure S2

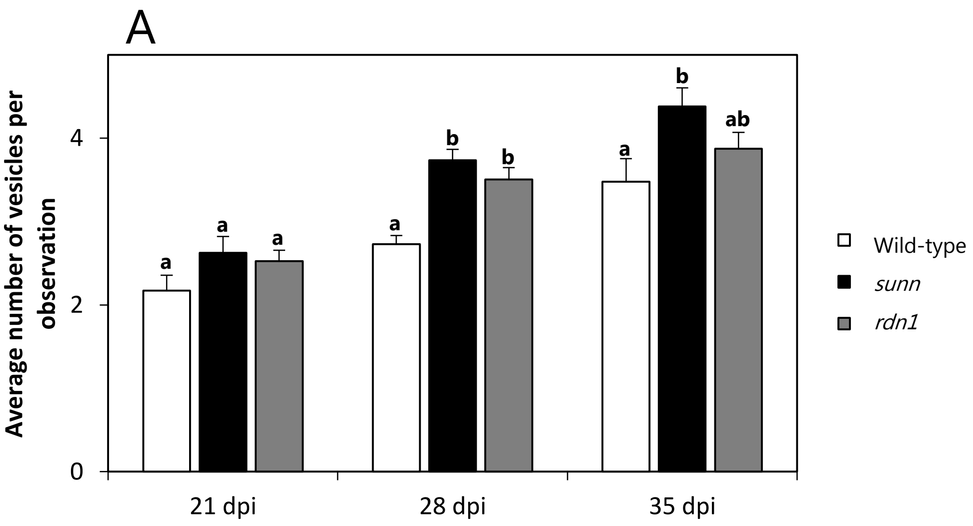
A
Average number of vesicles per observation
0
2
4
a
a
a
a
b
b
a
b
ab
21 dpi
28 dpi
35 dpi
Wild-type
sunn
rdn1

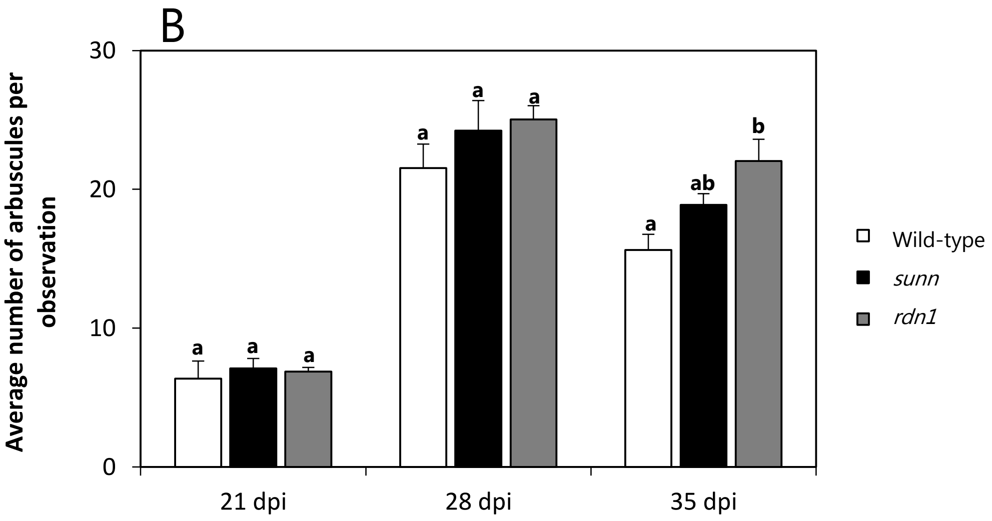
B
Average number of arbuscules per observation
0
10
20
30
a
a
a
a
a
a
a
ab
b
21 dpi
28 dpi
35 dpi
Wild-type
sunn
rdn1

Figure S3

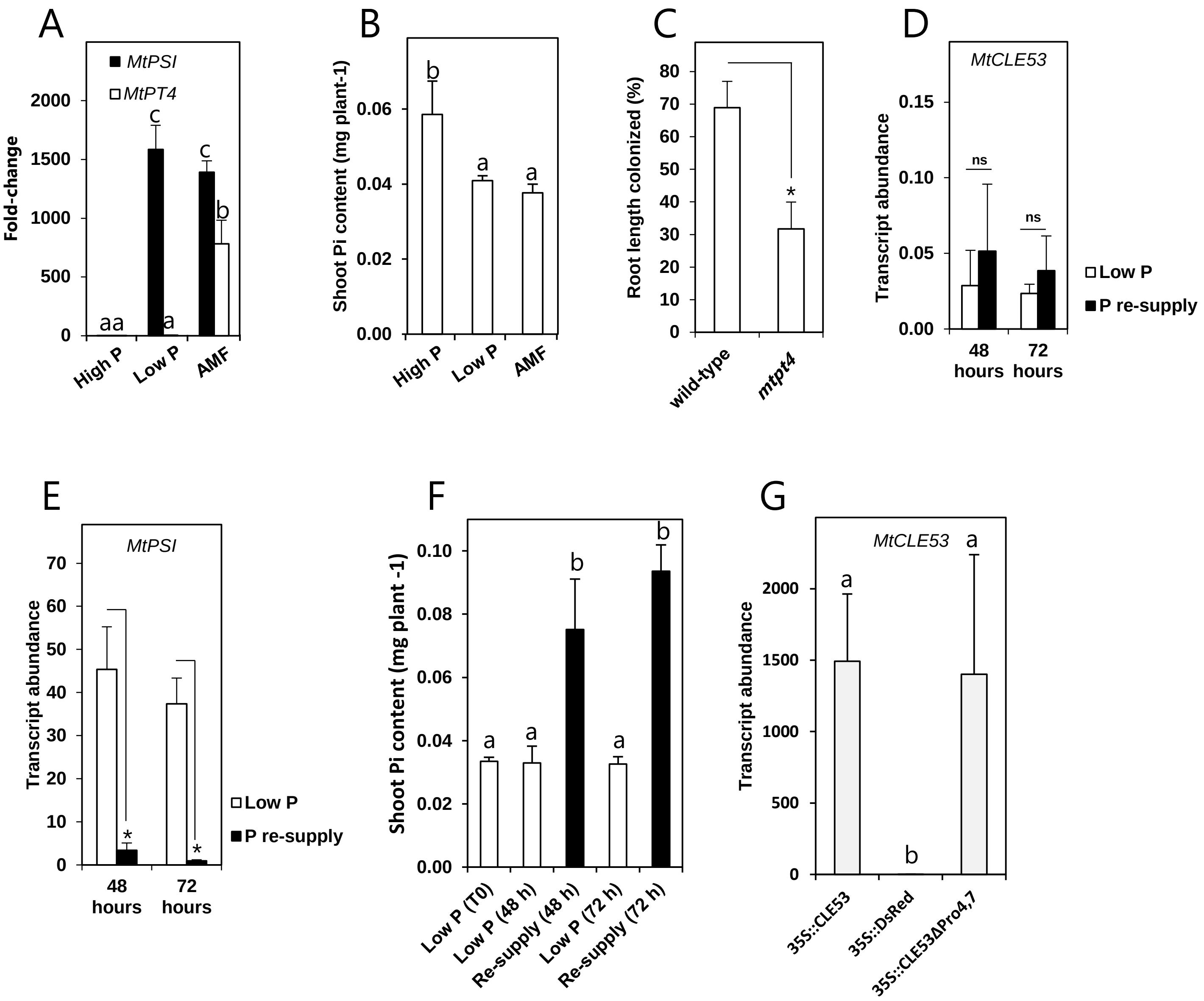
A
MtPSI
MtPT4
Fold-change
High P
Low P
AMF
B
Shoot Pi content (mg plant-1)
High P
Low P
AMF
C
Root length colonized (%)
wild-type
mtpt4
D
MtCLE53
Transcript abundance
48 hours
72 hours
Low P
P re-supply
E
MtPSI
Transcript abundance
48 hours
72 hours
Low P
P re-supply
F
Shoot Pi content (mg plant -1)
Low P (T0)
Low P (48 h)
Re-supply (48 h)
Low P (72 h)
Re-supply (72 h)
G
MtCLE53
Transcript abundance
35S::CLE53
35S::DsRed
35S::CLE53ΔPro4,7

Figure S4

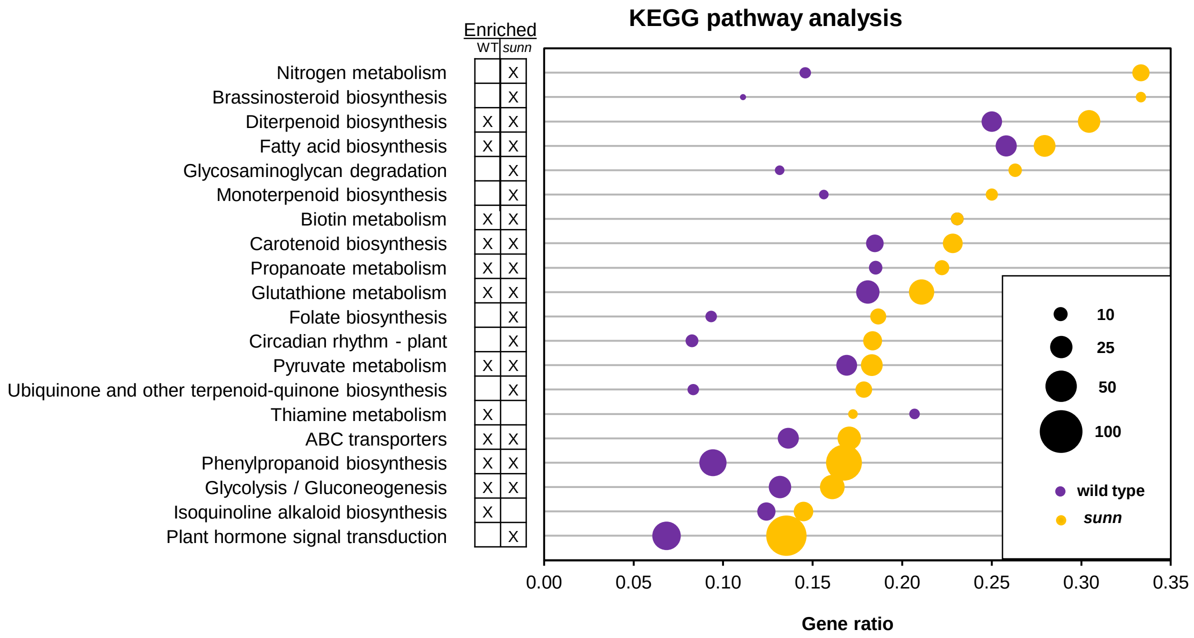
KEGG pathway analysis
Enriched
WT
sunn
Nitrogen metabolism
Brassinosteroid biosynthesis
Diterpenoid biosynthesis
Fatty acid biosynthesis
Glycosaminoglycan degradation
Monoterpenoid biosynthesis
Biotin metabolism
Carotenoid biosynthesis
Propanoate metabolism
Glutathione metabolism
Folate biosynthesis
Circadian rhythm - plant
Pyruvate metabolism
Ubiquinone and other terpenoid-quinone biosynthesis
Thiamine metabolism
ABC transporters
Phenylpropanoid biosynthesis
Glycolysis / Gluconeogenesis
Isoquinoline alkaloid biosynthesis
Plant hormone signal transduction
10
25
50
100
wild type
sunn
0.00
0.05
0.10
0.15
0.20
0.25
0.30
0.35
Gene ratio
